# Improving witness interviewing for the investigation of disappeared persons due to armed conflict

**DOI:** 10.1080/20961790.2021.2009626

**Published:** 2022-02-10

**Authors:** Derek Congram, Maria Mikellide, Matthew Vennemeyer

**Affiliations:** International Committee of the Red Cross, China

**Keywords:** Forensic sciences, missing persons, witness interview, humanitarian forensics

## Abstract

Investigating missing persons who are presumed dead in conflict contexts almost always involves witness interviews. Interviews can be critical both to locating burial sites and to positively identifying bodies that are located. Despite the importance of interviews, the authors have found that investigators in these contexts seldom have formal training on interviewing. This article highlights three principal problems that the authors have experienced relative to interviewing as part of missing persons investigations in armed conflict contexts: that interviewing is not treated as a professional activity; the tendency to overvalue technical tools in the search for burial sites; and a lack of awareness about cultural and contextual factors that impact interviews. The article concludes with five recommendations on how to improve witness interviewing.

Recently, staff from an international organization received a request from a government agency in Latin America requesting training on witness interviewing for the investigation of people, presumed dead, who went missing during armed conflict. The staff of the organization gave an immediate positive response, which triggered an internal debate: although some of the staff had significant experience interviewing witnesses in multiple active and post-conflict contexts, how did they know *if they did it well?* Doing something many times does not necessarily mean that the quality improves over time or that it is ever done well. In an age of increasing expert certification, how do we know what makes people experts at interviewing, and more specifically in the case above, if the organization’s staff do it better than the state agents who had requested training? A follow-up meeting between the two agencies resulted in a confession by one of the organization’s members, admitting that he questioned his own expertise as well as that of his colleagues, despite their significant experience interviewing. This inspired a plan for a joint training event for both groups by a panel of experienced professionals of different backgrounds with extensive interview experience and formal training: a veteran prosecutor, a tenured academic, and a forensic anthropologist who had worked at a research centre interviewing ex-combatants about disappearances.

This article is about key problems encountered by us, the authors, in the course of our investigations of missing persons in conflict contexts with diverse organizations. This article is motivated by a frustration with the lack of formal training about how to conduct productive interviews, but also by a desire to share our experience and make recommendations. Our skills are a product of lived experience and reflection about what has worked well and what has not in different situations. Considering that interviewing is such an essential element to investigations of missing persons, it is very surprising that more attention has not been given to the subject. Greater attention must be given to “lessons learned” and formal training to improve interviewing skills and avoid mistakes made. We have observed that knowledge acquired about the process of interviewing is seldom documented and passed on. In this way, we fail to treat interviewing as other scientific methods that require us to make observations, analyse results, recognize limitations, and share what we have learned.

This article is a review of witness interviewing as a key activity in the investigation of missing persons in conflict or post-conflict contexts. We will highlight problems that we have encountered throughout our professional experience in various countries and make several recommendations about effective witness interviewing. The principal problems we will address are:

1. Interviewing is a professional activity requiring developed skills but is seldom treated as such. Treating it as such means information-sharing/learning exchange, and formal training;
2. There is a tendency to view technical or “scientific” tools as more useful relative to information collected during interviews; and
3. Interview effectiveness is impacted by the context and conditions of the interview. To be effective, interviews should be planned and informed by both the context in which the interview takes place as well as the identities of the interviewer/interviewee.

## Interviewing as a professional activity

To most effectively elicit useful information through interviews, interviewing should be treated as a professional undertaking. Therefore, effective interviewing requires serious attention and training. We base this argument on our collective professional experience conducting investigations in the framework of different mandates. We have observed that interviewing witnesses, including both chance eyewitnesses and those people responsible for disappearance, is almost always treated as something that does *not* require specific training. Instead, interviews are perceived as something very “normal” and intuitive, an activity at which people are naturally proficient. It should be surprising that certain stages of processes as important as investigations of disappeared persons be treated as a common activity unworthy of professional training. Our experience has taught us that although interviewing can *seem* quite natural—almost synonymous with conversation—that does not mean that we are good at it.

At the most basic level, we have found that information about methods, practice, and “tips” are usually exchanged informally, often when an experienced person is being replaced by another person who is new to a context. Although this information exchange might be framed as part of a formal handover process, there is seldom time dedicated to serious contemplation about lessons and skills learned, what has worked and what has not. This is a limitation at the level of individual investigators, but the problem extends to organizations.

O’Brien and Kebbell [1] surveyed investigators and lawyers who worked for international tribunals and criminal investigative agencies about witness interviewing, noting that none of those surveyed had received any interview-related training at the institutions where they worked. They concluded that “it is important that the courts, particularly the ICC (International Criminal Court), adopt a comprehensive and court-wide training program” (p. 100).

Very recently, Sutliff and Severino [2] note that among police agencies in the US, there is “very little standardization, monitoring or validation of existing practices” (p. 3). The authors describe how in 2009, following controversy over US interrogations in Iraq and Guantanamo Bay, the authorities created a task force to study interrogation techniques that would be effective as well as consistent with the rule of law (e.g. not qualify as torture). This resulted in research collaboration with the Los Angeles Police Department that helped implement new, more effective, science-based interview techniques including advance planning, observation of linguistic, rather than behavioural clues, and the establishment of rapport with interviewees.

In early 2021, to address the problem of coercive interviews, which could include torture, and which often produce unreliable information, the Méndez Principles on effective interviewing were published. The fourth of these states that “[e]ffective interviewing is a professional undertaking that requires specific training” [3].

Recognizing the impact that interview method can have on an interviewee is also important when we consider that investigations of disappearances can be directly related to traumatic events. This introduces not only a responsibility toward the interviewee—to avoid exacerbating trauma—but also considerations about how memory works under these circumstances. Traumatic and negative experiences are less well-recalled than positive experiences with regard to some sensory information and some aspects of the narrative structure of the event [4–6].

There is a lot of academic research on interviewing, particularly in the social sciences [7–10]. Snook et al. [11] observed that in Canada interviewing methods in police contexts were improving due to practitioner–academic partnerships. Even still, the academic spirit of openly sharing research is not always so evident due to journal access rights, jargon, and social barriers that prevent exchange between academics and practitioners. Barriers exist at both ends of this potential exchange. Investigative agency protocols—if they exist—can be difficult to access. Investigative agencies may be protective or sensitive about their particular methods and unwilling to share “trade secrets”, or they might simply doubt the trustworthiness of academics. The concept of publishing and information-sharing that academics live by is often quite distinct from policies related to transparency of public security agencies.

There is an abundance of literature about interview methods, including the Strategic Use of Evidence (SUE) technique [12]; Human Intelligence Interrogations [13]; structured investigative interviews [14]; and serial interviewing [15], to name just a few. How many people who routinely conduct interviews as part of the investigation of disappeared persons in conflict contexts are aware of this rich body of literature? Treating interviewing as a professional activity means being aware of and informed by relevant literature as part of a knowledge base [16].

We have also observed a competitive tendency between governmental agencies; between governmental and non-governmental agencies; and particularly between non-governmental agencies, all of which prevents mutual learning and collaboration toward case resolution. Despite shared goals, organizations and agencies tend to compete for resources, attention, and individual successes, including the perhaps narrow-minded promotion of a particular method or organizational orientation. This competition can become more acute as investigations advance with time and success rates drop. This tendency of declining returns has been documented by Mikellide [17], where investigations begin with high resources and reasonably high success rates. As reliable witnesses become exhausted and the most obvious burial sites of missing people are discovered, success rates can drop dramatically [18,19]. With a decrease in successfully located burials, resources typically also are reduced, although one could argue that in fact they should increase, as at this point the work has become more challenging. This stage of increased challenges can foment unjust criticism and unhealthy competitiveness among agencies, prompting publicity campaigns that might exaggerate individual advances over coordinated success.

Regardless of whether the search is a multi-agency effort or an individual one, evidence collection and analysis often require specific instruments—ideally calibrated and tested for quality control—and standard operating procedures, as well as training on the proper interpretation of observations. Yet, this is not how witness interviews are often conducted. Of course, witness interviewing centres on human behaviour and memory, so is less controllable and quantifiable than many other types of forensic analysis. Nevertheless, treating witness interviewing in a similar way to other forms of evidence collection and analysis will help promote a greater respect for the activity as a procedure requiring skill that can elicit information (evidence) more productively.

Witness interviewing as a professional activity requires understanding existing pertinent literature and techniques, research, training, and continued development that question current models and their success. Ideally, all of this should be the product of collaboration among different agencies, as well as those in academia who are often better placed to conduct research that can test the reliability of particular interview methods.

## There is a tendency to view technical or “scientific” tools as more reliable than information collected during interviews

Although witness interviewing is such a basic, ubiquitous and often critical activity in the investigation of missing persons, a lot of popular and even institutional attention is given to high-tech search methods. In part this is fuelled by popular media, where technological solutions are perceived as both attractive and unfailing, solving cases within an hour (including commercial breaks). In contrast, a good, amicable, extended interview is seen as boring, except, perhaps, to social scientists and social workers.

One illustration of this relates to deaths of children at Indian Residential Schools in Canada. For over a century, children had been dying disproportionately at these schools due to poor living conditions and abuse [20]. A study of archives, interviews, and location visits showed that more than 4 000 children died at the schools and this was published in 2015 by a Truth and Reconciliation Commission [21]. Yet, it was not until 2021, when a ground penetrating radar survey was conducted at a burial site in Kamloops, Alberta, that the general public and media appeared to really believe in the deaths and the subject became a serious public concern. One headline read: “Technology uncovered remains at B.C. residential school but secrets still remain beneath the soil” [22]; another was: “This radar technology helped find the burial site of 215 children in Kamloops, B.C.—could it find others?” [23].

A second interesting example is from Cyprus where, as mentioned above, the successful location of burials has reduced dramatically in recent years. To help increase chances of grave discovery, Abate et al. [19] advocate a combination of digital technologies. Using two potential sites as case studies, they succeed in locating sub-surface anomalies, but not graves. The study not only demonstrates a limitation of digital technologies (i.e. cannot distinguish the nature of anomalies)—despite the investment of significant resources—but it also highlights the importance of reliable witness testimony as a starting point for their application. Tellingly, the authors discuss the absence of witness testimony on the depth of burials as a limitation for the application of such technologies while also noting the approach of the Commission for Missing Persons in Cyprus so far relative to witness interviews. They state that “…there has not been a conscious effort to locate and interview everyone who witnessed deaths and/or burials. Rather, testimonies have been provided spontaneously…as many witnesses are gradually passing away, interviewing/reinterviewing in order to collect more specific details that may aid in future searches is becoming increasingly difficult” (p. 97).

More than promoting digital solutions we argue that this case really illustrates how more time invested to train investigators on interview techniques and more time spent to plan, prepare, structure and analyse interviews, would be more productive and would have ensured that more testimony was recorded at the first stages of investigation.

It follows that more thorough interview techniques may have also helped in the application of new technologies as well as the discovery of more burial sites. The resources and time required for good quality witness interviews, including pre-interview preparation, the interview itself, and post-interview analysis, can be miniscule compared to the resources spent on the archaeological search—with or without digital and other remote sensing technologies—for a clandestine grave based on imprecise, incomplete, or inaccurate information.

On many occasions, we have been asked to speak to groups specifically about advanced technological methods to search for clandestine graves. At one of these, after speaking about ground penetrating radar, satellite imagery, and spatial analysis using geographic information systems, the speaker concluded saying that despite the potential and encouraging research on new technical methods, the large majority of clandestine graves that they successfully located were based exclusively on only two elements: good direct witness testimony and excavation with simple hand tools. A Commission tasked with finding and memorializing mass graves in Sierra Leone likewise reported that: “In gathering the desired information they [the Commission] relied largely on primary sources, i.e. people who were eye-witnesses to the killing and/or burial. It is the many interviews with these persons that led the investigators to the different sites” [24, p. 2]. Similarly, the location of mass graves in the former Yugoslavia relied principally on witness testimony [18, p. 91, 25, 26].

**Figure 1.**
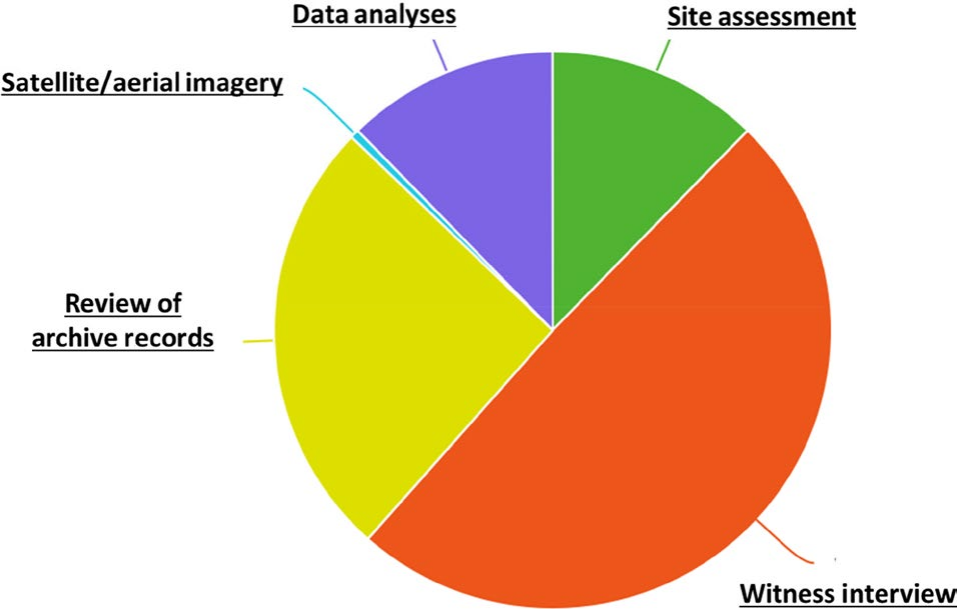
A theoretical distribution of the relative values of different activities related to the search for missing persons in conflict contexts.

There is often an unrealistic expectation that cases can be solved quickly and easily and that high-tech tools enable this. Figure 1 depicts the relative values of different activities involved in the search. It is a theoretical model, as different contexts offer distinct possibilities and so the weighting of each activity might be slightly different. What we want to emphasize with this is that witnesses provide the largest proportion of useful information. High-tech methods, which can be used as part of a site assessment of possible burials, in conjunction with data/mapping analysis or review of archive records, hold a proportionally smaller value (Figure 1).

Of course, experience tells us that the search is more complicated than this and there can be other factors that impact successful location of a burial site (such as whether or not the witness is telling the truth and has accurate and precise memory). However, we use this illustration to emphasize the disproportionate importance often placed on methods *other than* simple witness testimony.

A good example of struggling to find the right balance among different investigative methods is the 166-year search for crew and British ships the HMS Terror and HMS *Erebus*, which disappeared in 1848 during an attempt to find the Northwest Passage. Local Inuit testimony of the abandoned ships having been trapped in ice was documented many times over the years, including soon after their disappearance. Some of the challenges to identifying the location of the ships included distinct Inuit and English place names/understanding of the names of the missing people and place names (see also [27]); conceptions about location and precision; perceived contradictions among different witness testimonies, exacerbated by difficulties of amateur interpretation; distinguishing direct and indirect witnesses; as well as doubts about the reliability of Inuit testimony [28]. In 2014, after more than a century and a half of sporadic searching and a more concentrated 6 years of Canadian government-led searching with side-scan sonar, “Government of Nunavut team members found two artefacts from the Ugjulik [the Inuit place name] wreck that had been cached by some unknown Inuk”. The location was near where, in 1869, an Inuk named Inukpujijuk had indicated one of the Franklin expedition ships had sank. Based on the alignment of the witness testimony and the new physical evidence, the government search was redirected and soon after, HMS *Erebus* was located 11 m below the water surface [29].

**Figure 2.**
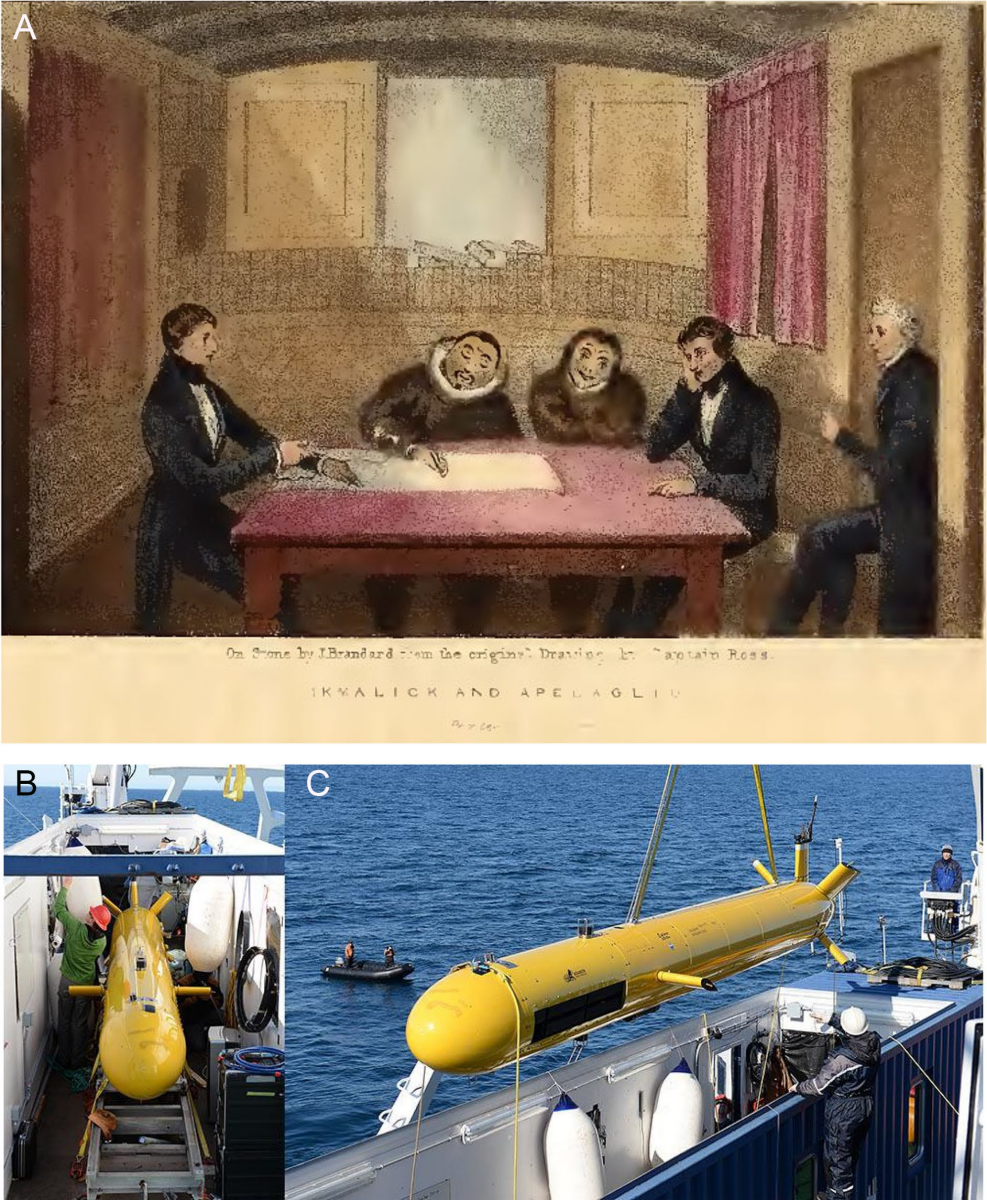
(A) Two Inuit, Ikvalick and Apelagliu, “very correctly” (in the words of Captain John Ross), map the Arctic landscape for Ross’ 1829 Arctic expedition, more than 15 years before the Franklin expedition. A depreciation of the value of witness testimony can lead to an unbalanced reliance on other methods, such as ground-penetrating radar or sonar (B and C), which can cause delay and add significantly to the cost of an investigation. Reproduced with permission from Nick Walker/Canadian Geographic and is from Ross [31] and Burke [32].

The reliability of Inuit representations of the landscape has been well documented, both historically and in modern times (Figure 2A) [30,31]. Nevertheless, the public at large and even investigative agencies are often more attracted to high-tech methods, such as sonar, in Figure 2 B and C.

By emphasizing the critical importance of direct witnesses and interviewing, we do not wish to discourage research on other technological methods. However, in many conflict contexts, these methods are impractical and more of a distraction than an aid. We feel that the professional literature related to technological methods is biased in favour of *success stories* and does not adequately acknowledge limitations or present an honest image of success *rates*. This happens both in research and practice: fruitful experiments are published in journals, and the successful location of clandestine burials are mapped and publicized in the professional and popular media. Negative findings do not receive the same treatment, despite having critical value in determining how we search for future burials and which tools we should use. A fundamental concept in science is that we learn from failed experiments, just as we do from successful ones; we learn not to repeat the failed experiment under the same conditions. Equally, it is also necessary to share inconclusive results (e.g. where there is not enough evidence to conclude the results support or refute a hypothesis, just as with an inconclusive determination of death during autopsy).

The success, then, to fairly weighting the value of witness testimony relative to other methods lies in attempting to quantify the true success rates of each respective method, something that can only be done by treating witness interviewing as a professional activity, by analysing and sharing the results of interviews, and by honestly sharing and assessing the results of other technological methods. Only once we have a clear understanding of the relative values of different methods based on a holistic understanding of how and under which conditions they each work best, can we avoid negative results based on attractive, but unpromising solutions.

## Interview effectiveness is impacted by the context and conditions of the interview

… scientific disciplines provide conditions in which actors working in different global contexts can claim a coherent professional identity and compatible epistemological assumptions despite very real variations in the social, cultural, and historical contexts in which science is actually practiced … scientific practice must be understood in local contexts. [33]

Asen, drawing on Edmond [34], conveys the balance required in applying the shared characteristics of a scientific practice across very distinct places, where practice might need to be adjusted to achieve the same goals in different situations. Getting the same evidence in diverse conditions might require adapted methods. This is especially true when that evidence is not physical, but testimonial.

Some studies have shown that cultural differences *within* countries can have an impact on information gleaned and so interview methods should be adapted depending on the nature of interviewee culture [35,36]. A very recent review of an investigation by the Toronto Police Service of serial homicide of gay men showed that investigators had trouble effectively communicating with the LGBTQ2+ community [37]. It stands to reason that the culture gaps and interview impact can be even greater when the interviewer and interviewee are from different countries.

After having worked in many different conflict contexts, often as foreign investigators, we have concluded that an essential element to effective interviews in the investigation of missing persons is that they are culturally informed. This sentiment is supported by recent academic literature. Hope and Gabbert [38] note that “there have only been limited attempts to evaluate the role of culture in the conduct of effective investigative interviewing with victims and witnesses” (p. 145). For a fascinating experimental exception, see Anakwah et al. [39]. Interviewing in conflict contexts is often done in places where investigators are culturally distinct from those being investigated and so socio-cultural differences can create obstacles. Obvious examples include when people from resource rich countries associated with past colonialism, are conducting interviews in resource poor countries, which had been colonised.

We also see these challenges in the search for the crew of the HMS *Erebus* and Terror through interviews of Inuit. From 1878–1880, former American soldier William H. Gilder [40] was on a search expedition. About speaking to Inuit witnesses, he said: “They have little idea of numbers beyond the number of their fingers, and such as they can borrow by calling attention to their neighbors’ fingers. Any sum that calls for more than that is to them amasuet (many) or amasuadelo (a great many)”. This was a problem in trying to account for sightings of those among the 128 crew members from the ships.

By the term “culturally informed”, we wish to emphasize the essential need to incorporate knowledge of local customs, manners, and context in the interview process, both for questioning and for interpreting responses. This element is only addressed briefly in the recommendations on Effective Interviewing mentioned earlier, which are meant to establish “international standards” [3]. These authors only fleetingly mention that certain characteristics of an interviewee can make them particularly vulnerable (p. 24). To be clear, employing methods and analysis that are culturally informed is not necessarily contradictory to having international standards; we do not suggest that interviewing is completely culturally relative. The challenge is defining the importance of each. International standards can refer to a minimum standard of interviewer behaviour that is required—for example, that interviews not be physically violent or coercive—yet, the interviewer can also adjust the place and form of an interview in a way that is more accommodating to the interviewee’s culture and, in effect, more conducive to eliciting relevant and reliable information in a non-coercive way.

Many so-called international investigations have called upon investigators who were effective in their own countries, however their effectiveness was limited when conducting interviews in countries, languages and cultures that were foreign to them. We have observed—and lived—the frustration that comes from failure to understand the intended meaning of words or even concepts that are easily translated, but not effectively interpreted or understood between interviewers and interviewees. This can include concepts of time, distance, and the related understanding of maps as representations of space, in addition to actual words and their meanings.

Culturally informed interviewing implies partnerships with those who understand the local culture, social dynamics, history, and politics. These partners can help formulate effective questions, advise on the most appropriate setting and conditions for interviews and assist with analysis and interpretation of responses.

In forensic investigation, we think often of Locard’s Principle of Exchange to help us understand how the perpetrator of a crime interacts (exchanges) with a scene and a victim. A similar dynamic applies during an interview, but in a social way: there is an exchange whereby the interviewer—by their appearance, identity, words, actions—influences the interviewee *and vice versa*. This action is not just a one-way or even two-way interaction, but an activity that is impacted by each of the participants as well as their setting. Environmental factors that might impact testimony include *who else* is present during an interview as well as the setting of the interview.

One of us was leading a team that was searching for the remains of a foreign military pilot whose plane had crashed near a village decades before the search. Arriving at the village on the first day, there were formal exchanges between the commission of officials from the host country and those from the country of the pilot. A woman was presented as an official witness and she related her testimony in front of all the officials. The woman claimed that the plane exploded for an unknown reason and that villagers ran to the crash site. According to the witness, the body of the pilot was wrapped in a white cloth, placed in a deep two-by-one-meter grave, a candle was lit, and a solemn ceremony was held while burying the body.

The witness indicated the location of the grave and excavations started the following day. After approximately 2 weeks of unsuccessful excavation by a team of approximately 30 people, another witness approached the investigation team leader. It was following a long lunch break and the officials of the host country either had not returned from lunch or were resting in hammocks in the shade. The new witness asked the investigator: “Do you want to know what *really* happened?” He started his story quietly, his volume and enthusiasm increasing as the story progressed. At the time of the crash, he had been in the army of the host country and operated a 57-mm anti-aircraft gun. He said that the pilot had been bombing the area. He pronounced proudly that it was he who had shot the plane down, after which he rushed to the crash site, celebrating. Arriving at the site, he found only a part of the pilot’s head, still in the helmet, which he pushed into a shallow depression in the ground and then covered it with a small amount of loose soil. He noted that different fruit trees had, over the subsequent decades, been planted across the area and he was certain that the investigators would not find anything.

This latter testimony seemed much more logical and explained why, after 2 weeks of searching, nothing had been found. In this case, the conditions of the interview had changed, and rather than a witness being nominated and presented to foreign officials in a tense, formal situation, this witness chose to give testimony privately and voluntarily, which was much more consistent with the evidence (or lack thereof).

Not only were the context and the conditions of the interviews important, but gender might also have been a factor. The first witness was the only woman among approximately 20 military and political personnel from both countries. How might being a female villager in a male-dominated society, surrounded by men of higher social status have impacted her testimony? The second witness, aside from being free of an audience of different high-ranking officials, was a man and a military veteran. In telling his story, he seemed to delight in recounting about how he successfully shot the pilot, the “enemy”, down. In that particular setting and under those conditions, it is understandable why he felt the way he did and wanted to tell his story. Although the conditions of this second testimony and the man’s identity do not guarantee the reliability of his account, there is good reason to trust him.

Interpreters are often required for interviews in these contexts. Translators typically provide direct, more-often literal written translation. Interpreters generally incorporate simultaneous oral translation with context, focused on meaning, rather than just literal words. Language, as a cultural product, is embedded with meaning that is represented in multiple ways, not only by the actual, technical words, but also in *how* they are spoken, *where* they are spoken, and by actions (body language). Police interviews, or interrogations, conventionally rely on behavioural (nonverbal) clues: does the interviewee seem nervous? Defensive? Do they avert their eyes when asked certain questions? These behaviours are often believed to indicate that someone is being untruthful or evasive, though studies show that this is misguided [41,42]. The interpretation of non-verbal behaviour can very much depend on cultural characteristics of the interviewee and context, as when eye contact is considered aggressive or rude, rather than indicate dishonesty.

In another case, an interpreter advised the investigator that it would be necessary to sit down and have tea with the family of a missing person before interviewing them. The interviewer objected, they had many interviews to conduct and several sites to visit that day. Sitting down to have tea with each family would be a loss of limited time. In fact, the local interpreter explained, the time invested in this social ritual was a show of respect for local culture as well as for the family, which was struggling with the ambiguous loss of a loved one. Time invested with the family at the beginning of an investigation could also have pay-offs in terms of the quality of information received. Poor information gathering during a rushed interview, where the interviewer shows a perceived disinterest in carefully discussing with the family the circumstances and characteristics of their loved one, may well require a second interview to get missing information or clarify doubts. Sitting down to have tea is far less about having tea, than it is about social relations, building rapport, and demonstrating empathy, which in turn creates conditions favourable to a productive interview. Even if the search does not immediately prove to be successful, at least both the investigator and the family have the confidence that a good effort was made and that the explanation is less likely attributable to a lack of due diligence. Of course, demonstrating respect has value in and of itself, and should not be perceived merely as means to an end.

Establishing good rapport with interviewees can help secure future collaboration [43]. This is particularly important in contexts where armed conflict is ongoing, but also for large organizations that have multiple lines of work to assist affected populations. It might be particularly challenging in conflict contexts where there is pressure on witnesses not to share information, added to the possible complication of doing so with a foreign investigator (who might be perceived, perhaps correctly, as not understanding the context, of being unfairly judgmental). However, we have also observed the *opposite* effect of the outsider-culture interviewer, where the interviewee is more inclined to share information because they believe the interviewer has an unbiased opinion.

Local interpreters might also have knowledge that can be useful for technical aspects of a search, even though they are not forensic subject matter experts. Local farmers, for example, are often very capable at making observations about historical and seasonal changes to landscapes, about native and invasive vegetation and for noting unnatural topographical or soil changes that might indicate a clandestine grave. They might observe that when looking from the nearest road a burial site is obscured from sight when deciduous trees have leaves, but the site is visible during cold or dry seasons when trees have lost their leaves.

Local interpretation can be useful for very simple, pragmatic things such as testimony related to time or distance. A two-hour walk to a site for a rural, indigenous person can easily be a four-hour slog for an out-of-shape foreign investigator. The extra one or two hours allocated for drinking tea and talking in greater detail can easily save many hours during the follow-up investigation and search.

There are many considerations that need to be made about interpreters as their identity (e.g. name, accent) and presence can impact—positively or negatively—the quality of responses from an interviewee. To be concise, we simply mention this as something for investigators to reflect upon as they prepare for, conduct, and analyse interviews.

Another important element related to culturally informed interviewing and language is terminology related to the dead, whether or not people who are missing are actually dead, as well as how to refer to them [44]. In the first case, it is very important to use language that does not mistakenly presume the missing are dead, as this can be very disheartening for families who are seeking missing loved ones. Here one must be honest (e.g. when good evidence strongly supports the hypothesis that the missing are dead), but clear, tactful and empathetic in the face of the suffering of families who might have confronted a probable death, but who might feel guilty about accepting that their loved one has died. The words that one uses to refer to a dead body can be sensitive. This has been a discussion in some countries in Latin America, for example, where some families of the missing object to the use of the word “human remains” (*restos humanos*) and prefer “bodies” (*cuerpos*). Complicating the situation, different state institutions might use distinct terminology, for example one agency could refer to “unidentified bodies”, while another uses “unidentified people”. These subtle differences might seem like an inane semantic debate for the foreign forensic investigator, but they can have great meaning for families of the disappeared. Humanizing the remains of victims (e.g. “person”) is important to the families and terms such as “cases” or “body” might seem bureaucratic and insensitive.

**Figure 3.**
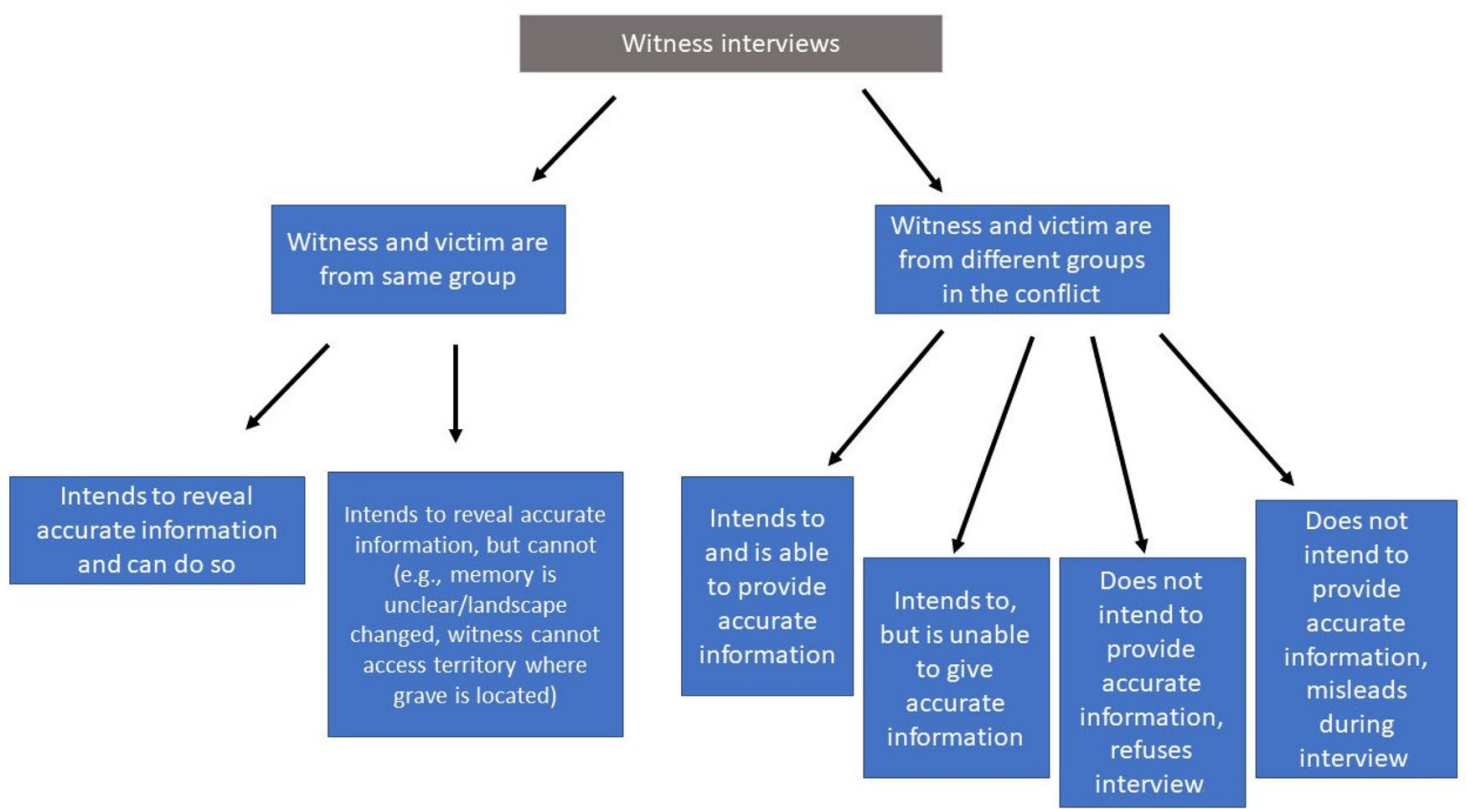
Witness interview schema showing different scenarios related to willingness and ability to provide information about missing person burial sites. This is an idealized (simplified) schema for a conflict between two groups, recognizing that people do not fall simply or exclusively into one or another group.

When we consider testimony about the location of graves and identifying missing persons, we must consider that this information can imply political capital for witnesses on both (or many) sides of a conflict. On the victim side, when the state or community resolves a case, they remove a name from the list of missing persons and register a new casualty, which might carry less (or more) symbolic value than a missing person [45]. For someone on the side of a group responsible for a disappearance, the informant may reveal sensitive information linked to legal violations that “their” side to the conflict may not want to be exposed. Both sides may be resistant to locating graves and clarifying the fate of the missing for different reasons, and witness testimony should always be understood within a broader socio-political framework. Therefore, the starting point of examining witness testimony should consider the situation of the person providing testimony: possible group affiliation, their role in the conflict, and their role in society at the time of the interview. There are different typologies of witnesses, which impact their testimony (Figure 3).

Interview dynamics relate to the identity of the interviewee relative to a person who has disappeared. Understanding willingness and ability of witnesses to provide useful information can help interpret responses. For example, accurate identification of a burial site may depend on access of the witness to the place. Their intention to do so may depend on the specific incentive of the individual and a community to reveal information. In particular, group cohesion or solidarity may impact the willingness or ability of the witness to provide useful information.

This final point about the cultural and situational complexities of interviews might help explain the first two problems we discussed in this article: that interviewing must be treated as a professional activity and the tendency to favour technical solutions over interviewing during investigations. A natural tendency when confronting something complex is to look for a simple solution. While this might be completely reasonable in some situations, we believe that the alternate solutions being used—treating interviewing as simply intuitive, relying exclusively or excessively on technical tools to search for graves, and copy-pasting interview protocols from one context to a very different one—are inadequate. As US journalist H. L. Mencken has famously said: “Every complex problem has a simple solution that doesn’t work.”

Considering all of the above, we make the following recommendations:

First, considering that witness interviewing is a professional activity of significant gravity in the investigation of missing persons in conflict contexts, we reiterate the call of O’Brien and Kebbell [1] to implement formal interview training. Although these authors made reference only to international investigators focused on suspect interviews and with a focus on criminal accountability mandates, we argue that this need also includes investigations on missing persons with a humanitarian focus.

Training should include how to conduct empathetic interviews of witnesses or victims, which is critical to try to mitigate the harm that revisiting traumatic events can provoke, just as is understanding how to best aid witness recall. We all recognize that in collecting information through interviews, investigators are trying to do good. What we sometimes fail to recognize is that the process of “doing good” can do harm. In saying this, we want to recognize that things are not so simple as the oft-cited, though naïve expression “do no harm” [46]. Many interventions—be they medical (e.g. chemotherapy), forensic (e.g. autopsy), or interviewing people about traumatic events—can be harmful. The goals of a professional interview should be to determine if that harm is acceptable given the overall goal and how to mitigate potential harm. This is not a simple thing, and doing it well requires professionalism.

Second, to overcome the common lack of knowledge/expertise transfer, we recommend implementing a review process that takes place following interviews. Just as with other scientific methods, there are concerns about quality control and quality assurance. Interviewing might not lend itself to very specific or rigorous protocols and, in fact, this might even be counterproductive as when cultural conditions demand adaption. However, establishing a process of review serves a similar purpose to peer-review and promotes a more thorough analysis of information obtained, ensures that possible missing information can be identified and sought, or contradictory testimony can be rectified, and that the investigator has a second or third opinion about interpretations of testimony.

Third, this last aspect should involve someone familiar with local culture so as to detect any nuance that might be missed by a foreign investigator, for example. There is a lot of nuance in assessing what a person’s words *really* mean, how they express themselves, and what might impact their willingness and capacity to share useful information. Crucially, in some cultures, interactions with foreigners and sharing any information with foreigners requires the permission of the local leaders. Without local partnerships it is unlikely that investigators will obtain reliable information from local communities who hold valuable local knowledge be it on locations of interest or conditions for search. Local knowledge can validate an interpretation or provide an alternative hypothesis based on witness testimony. This recommendation is more specific than, but in line with one of the recommendations made by Baraybar et al. [47] on investigations in general.

It follows that we advise to give *time* for a thorough investigation to take place rather than a speedy investigation which quickly leads to excavations in the search for burial sites. Experience has shown than a poor investigation leads to longer periods of excavations with often no results, which can quickly drain financial sources and prevent more reliable cases from being explored. To this effect it “pays” to have a thorough investigation which can offer reliable leads limiting the area to be explored and the time required.

Fifth, continuity in investigations is critical to enable all the above to take place. The authors’ personal experience in multiple contexts have shown that staff turnover has damaging consequences for investigations. This is demonstrated through searches in locations already explored, inability to cross-reference information and link pieces of information as they get lost during transitions and most importantly an inability to build trust with communities and understand local contexts.

## Conclusion

Interviewing for investigations of missing persons is an essential, almost ubiquitous starting point. In our experience, we have observed that many people who conduct these types of interviews in active or post-conflict contexts have little or no formal training. Considering that so much critical information comes from interviews of witnesses of disappearances, it is remarkable that so few organizations have formal training programmes for investigators.

Interviewing is a professional activity. Treating interviewing as a professional investigation tool means studying past research and protocols, as well as documenting, analysing, and sharing lessons learned just as we do with other analytical techniques. There is a rich body of academic and professional literature as well as accumulated lived experience among veteran investigators. Organizations that sponsor investigations should ensure formal development and continuity of skills.

As interviewing becomes more widely recognized as a professional activity, its relative value when compared with other investigative tools will be better weighted. Professionalizing interviewing will also help lawyers, courts, affected populations, funding agencies, and the public at large have a more realistic appreciation of the relative utility of complimentary technical methods, which can be impractical or even useless in some contexts. High-tech methods will continue to be developed and we applaud this, but the principal challenges are evaluating the true utility of existing methods in particular situations with unique conditions and validating new methods.

Part of the process of professionalizing interviewing involves learning how to adapt the interview to distinct contexts. This can involve serious considerations of language, investigator/interpreter/interviewee identity, the political environment, local history, and culture, among other things. Our experience has taught us that even the most experienced investigator can perform poorly if she or he is working in an unfamiliar environment. Partnering with people who have local knowledge is important in preparing for and conducting interviews, as well as reviewing the information acquired during an interview.

Sceptics of the true value of interviews and the professionalization of the process might dismiss our recommendations due to the time, money, institutional changes, and personnel hours that they require. However, we are certain that these changes represent an investment in more efficient and effective interviews. Four hours invested in interview preparation, execution, and analysis can save 4 weeks of excavation at an alleged burial site based on unreliable information. Money saved at the beginning of investigations, when witnesses are more abundant and more burial sites exist to be located, can be used later when returns on witness information diminish.

## References

[1] O’Brien M, Kebbell M. Interview techniques in international criminal court and tribunals. In: Bull R, editor. Investigative interviewing. New York (NY): Spring Science + Business Media; 2014.

[2] Sutliff U, Severino M. The art of the inquiry: the LAPD’s journal with science-based interview techniques. Center for Cyber and Homeland Security at Auburn University; 2019. Available from: http://www.jstor.com/stable/resrep20760

[3] Méndez JE, Thomson M, Bull R, et al. Principles on effective interviewing for investigations and information gathering. Tri-institutional coordination of the Association for the Prevention of Torture, the Center for Human Rights & Humanitarian Law of the American University, and the Norwegian Center for Human Rights at the University of Oslo; 2021.

[4] Byrne CA, Hyman IE, Scott KL. Comparisons of memories for traumatic events and other experiences. Appl Cognit Psychol. 2001;15:S119–S133.

[5] Mladina V. Psychosocial aspects of interviewing and self-care for practitioners. In: Congram D, editor. Missing persons: multidisciplinary perspectives on the disappeared. Toronto (Canada): Canadian Scholars’ Press Inc; 2016.

[6] Southwick SM, Morgan CA 3rd, Nicolaou AL, et al. Consistence of memory for combat-related traumatic events in veterans of Operation Desert Storm. Am J Psychiatry. 1997;154:173–177. 9016264 10.1176/ajp.154.2.173

[7] Fontana A, Prokos AH. The interview: from formal to postmodern. Walnut Creek (CA): Left Coast Press; 2017.

[8] Deeb H, Vrij A, Hope L, et al. The devil’s advocate approach: an interview technique for assessing consistency among deceptive and truth-telling pairs of suspects. Leg Crim Psychol. 2018;23:37–52.

[9] Filipović L. Police interviews: communication challenges and solutions. PS. 2019;10:1–8.

[10] Salmon K, Pipe ME, Malloy A, et al. Do non-verbal aids increase the effectiveness of ‘best practice’ verbal interview techniques? An experimental study. Appl Cognit Psychol. 2012;26:370–380.

[11] Snook B, Eastwood J, Barron WT. The next stage in the evolution of interrogations: the PEACE model. Canadian Crim Law Rev. 2014;18:219–239.

[12] Granhag PA, Strömwal PA, Hartwig M. The SUE technique: the way to interview to detect deception. Forensic Update. 2007;88:25–29.

[13] Hartwig M, Meissner CA, Semel MD. Human intelligence interviewing and interrogation: assessing the challenges of developing an ethical, evidence-based approach. In: Bull R, editor. Investigative interviewing. New York (NY): Spring Science + Business Media; 2014.

[14] Simons AB, Boetig PB. The structured investigative interview. FBI Law Enforc Bull. 2007;76:9–20.

[15] Read BL. Serial interviews: when and why to talk to someone more than once. Int J Qual Methods. 2018;17:1–10.

[16] Passalacqua NV, Pilloud MA, Congram D. Forensic anthropology as a discipline. Biology. 2021;10:691–707. 34439924 10.3390/biology10080691 PMC8389313

[17] Mikellide M. Recovery and identification of human remains in post-conflict environments: a comparative study of the humanitarian forensic programs in Cyprus and Kosovo. Forensic Sci Int. 2017;279:33–40. 28837886 10.1016/j.forsciint.2017.07.040

[18] Sarkin J, Nettelfield L, Matthews M, et al. Bosnia i Herzegovina; missing persons from the armed conflicts of the 1990s: a stocktaking. Sarajevo (Yugoslavia): International Commission on Missing Persons; 2014. Available from: https://www.icmp.int/wp-content/uploads/2014/12/StocktakingReport_ENG_web.pdf

[19] Abate D, Sturdy Colls C, Moyssi N, et al. Optimizing search strategies in mass grave location through the combination of digital technologies. Forensic Sci Int Synerg. 2019;1:95–107. 32411961 10.1016/j.fsisyn.2019.05.002 PMC7219194

[20] Maass A. Perspectives on the missing: residential schools for aboriginal children in Canada. In: Congram D, editor. Missing persons; multidisciplinary perspectives on the disappeared. Toronto (Canada): Canadian Scholars Press; 2016. p. 13–40.

[21] Honouring the truth, reconciling for the future; summary of the final report of the Truth and Reconciliation Commission of Canada; 2015. Available from: https://nctr.ca/records/reports/

[22] Hua J, Chernecki T, Azpiri J. Technology uncovered remains at B.C. school but secrets still remain beneath. Global News. Available from: https://globalnews.ca/news/7909761/ground-penetrating-radar-kamloops-residential-school/

[23] Boyd A, McKeen A. This radar technology helped find the burial site of 215 children in Kamloops, B.C. — could it find others? Toronto Star; 2021. Available from: https://www.thestar.com/news/canada/2021/06/01/this-radar-technology-helped-find-the-burial-site-of-215-children-in-kamloops-bc-could-it-find-others.html

[24] Sierra Leone CA. Truth and Reconciliation Commission final report, Appendix Four: memorials, mass graves and other sites; 2004. Available from: http://www.sierraleonetrc.org/index.php/view-the-final-report

[25] Manning D. Srebrenica investigation: summary of forensic evidence — execution points and mass graves. Report to United Nations International Criminal Tribunal for Former Yugoslavia, 16 May 2000.

[26] Komar D. Patterns of mortuary practice associated with genocide: implications for archaeological research. Curr Anthropol. 2008;49:123–133.

[27] Congram D, Kenyhercz M, Green AG. Grave mapping in support of the search for missing persons in conflict contexts. Forensic Sci Int. 2017;278:260–268. 28787668 10.1016/j.forsciint.2017.07.021

[28] Woodman DC. Unravelling the Franklin mystery. 2nd ed. Inuit Testimony. Montreal (Canada): MQUP. 2015.

[29] Parks Canada, online. Wrecks of HMS *Erebus* and HMS Terror national historic site. 2021. Available from: https://www.pc.gc.ca/en/lhn-nhs/nu/epaveswrecks/culture/inuit/qaujimajatuqangit

[30] Rundstrom RA. A cultural interpretation of Inuit map accuracy. Geog Rev. 1990;80:155–168.

[31] Ross J. Narrative of a second voyage in search of a North-West passage. London (UK): Webster. 1835.

[32] Burke T. Queen Maud Gulf the focus of the search — for now. Canadian Geographic. 2014. Available from: https://www.canadiangeographic.ca/article/queen-maud-gulf-focus-search-for-now

[33] Asen D. Death in Beijing: murder and forensic science in Republican China. Cambridge (UK): Cambridge University Press; 2016.

[34] Edmond G. The law-set: the legal-scientific production of medical propriety. Sci Technol Human Values. 2001;26:191–226.

[35] Chenier K, Milne R, Shawyer A, et al. Police victim and witness interviewing in a Northern Canadian territory: measuring perceptions and practice. J Police Crim Psych. 2020. doi: 10.1007/s11896-020-09417-8

[36] Eades D. Understanding aboriginal English in the legal system: a critical sociolinguistics approach. Appl Linguist. 2004;25:491–512.

[37] Epstein GJ. Missing and missed: report of the independent civilian review into missing persons investigations. Executive summary and recommendations. 2021;1. Available from: https://tpsb.ca/component/jdownloads/category/61-missing-and-missed

[38] Hope L, Gabbert F. Interviewing witnesses and victims. In: Brewer N, Douglass AB, editors. Psychological science and the law. New York (NY): Guilford Publications; 2019. p. 130–156.

[39] Anakwah N, Horselenberg R, Hope L, et al. Cross-cultural differences in eyewitness memory reports. Appl Cognit Psychol. 2020;34:504–515.

[40] Gilder WH. Schwatka’s search; sledging in the arctic in quest of the Franklin records. London (UK): Sampson Low, Marston, Searle, and Rivington; 1882.

[41] Hartwig M, Granhag PA, Strömwall LA, et al. Police officers’ lie detection accuracy: interrogating freely versus observing video. Police Q. 2004;7:429–456.

[42] Snook B, McCardle MI, Fahmy W, et al. Assessing truthfulness on the witness stand: eradicating deeply rooted pseudoscientific beliefs about credibility assessment by triers of fact. Canadian Crim Law Rev. 2017;22:297–306.

[43] Pounds G. Rapport-building in suspects’ police interviews: the role of empathy and face. Pragmat Soci. 2019;10:95–121.

[44] Salado Puerto M, Abboud D, Baraybar JP, et al. The search process: integrating the investigation and identification of missing and unidentified persons. For Sci Int Synerg. 2021;3:1–22. 10.1016/j.fsisyn.2021.100154 PMC8219753 34189449

[45] Kovras I, Loizides N. Delaying truth recovery for missing persons. Nations Natl. 2011;17:520–539.

[46] Terry F. Condemned to repeat? The paradox of humanitarian action. Ithaca (NY): Cornell University Press; 2002.

[47] Baraybar JP, Brasey V, Zadel A. The need for a centralized and humanitarian-based approach to missing persons in Iraq: an example from Kosovo. Int J of Human Rights. 2007;11:265–274.

